# New psychometric evidence for the thesis advisor abuse scale (EMAT) in Peruvian university students based on classic and modern procedures

**DOI:** 10.1016/j.heliyon.2024.e28475

**Published:** 2024-03-20

**Authors:** Oscar Mamani-Benito, Maria Elena Rojas-Zegarra, Renzo Felipe Carranza Esteban, Tomás Caycho-Rodríguez, Lindsey W. Vilca, Susana K. Lingán-Huamán

**Affiliations:** a Facultad de Ciencias de la Salud, Universidad Señor de Sipán, Chiclayo, Perú; b Escuela de Posgrado, Universidad Nacional de San Agustín de Arequipa, Arequipa, Perú; c Carrera de Psicología, Facultad de Ciencias de la Salud, Universidad San Ignacio de Loyola, Lima, Perú; d Facultad de Psicología, Universidad Científica del Sur, Lima, Perú; e South American Center for Education and Research in Public Health, Universidad Norbert Wiener, Lima, Perú

**Keywords:** Mistreatment, Thesis advisor, Psychometric research, Perú

## Abstract

Although evidence of mistreatment toward university students in the academic field has been reported for several years, its study in the context of the development of undergraduate research is still emerging. For this reason, it is necessary to have valid and reliable measurement instruments that allow assessing the magnitude of this problem. The objective of this study was to evaluate the psychometric properties of the Thesis Advisor Abuse Scale (EMAT, for its acronym in Spanish) in Peruvian university students. A total of 753 university students (women = 57.4%) from the 3 regions of Peru participated. The internal structure was analyzed under an analytical-factorial approach, and the discrimination and difficulty characteristics of the items were evaluated from the perspective of item response theory (IRT). The findings showed evidence supporting the original three-dimensional structure. Furthermore, all the items on the EMAT have good discriminatory power. Additionally, the EMAT proved to be strictly invariant according to sex, and the reliability coefficients reached high magnitudes. It is concluded that the EMAT is an instrument that has adequate psychometric properties to be used as a measure of mistreatment by advisors in the thesis preparation processes in Peruvian university students.

## 1 Introduction

Mental health in the context of university research is an understudied topic. Based on the evidence found, there are certain attitudes and behaviors on the part of thesis advisors, evaluating juries, and administrative staff in charge of managing the documentation and the respective procedures, which alter the emotional health of students who carry out research work aimed at obtaining academic degrees and titles [1].

When reviewing available scientific literature, one observes that mistreatment in higher education is a topic that has been discussed for many years [[2], [3], [4], [5]]. However, in research contexts, the information is very scarce. For example, in Peru, some studies characterized the most frequent types of abuse [3] as stress due to the perception of violence during medical training [6] and alterations in mental health, such as depression, associated with receiving mistreatment during pre-professional practices [7].

At the international level, it was also possible to corroborate that the university population is vulnerable to receiving some abuse during their professional training [[8], [9], [10]], as revealed in studies carried out in Colombia [8], Mexico [10] and the United States, a country where the foundations and realities of ‘public humiliation’ in the training of medical professionals were thoroughly investigated [11]. Thus, there are reports where medical students [12], interns who completed rotations in internal medicine, surgery, pediatrics, obstetrics, gynecology, and neurology [13], and interns who had completed their surgical internship [14], revealed having been victims of mistreatment by their teachers and medical supervisors.

An important point to highlight as a result of this review is the difference in the approach to the problem between underdeveloped countries and first-world countries, such as the United States, where alternatives are observed to mitigate the problem of abuse in the academic field. , applying intervention programs to develop a safe environment so students can report teacher mistreatment [15]. A group of researchers led by Lind et al. [16] implemented interventions over 6 years that helped reduce the rate of student-reported mistreatment by 36% compared to a 4% decrease across all US medical schools.

Focusing the analysis on the primary variable, mistreatment of the thesis advisor is defined as an intentional manifestation of power and imposition of criteria, which include verbal insults, intimidation, abuse, and contempt, in addition to the act of ignoring or rejecting some work or request from the thesis student [17]. Although there is still no complete theory to explain this phenomenon, the literature accounts for two perspectives with which the attitudes and behaviors of some advisors, juries, and administrators could be explained to the detriment of students who prepare their theses. Firstly, academic authoritarianism is a construct where non-dialogical communication, hegemonic relationships, and punitive evaluation predominate. That is, it happens when a teacher abuses his authority to subject the student, generating a perception that he is the owner of the knowledge and the student is a passive recipient, in some cases using evaluation as an instrument of punishment [18].

About this theme, León [19] comments that authoritarianism has become a collectivist evaluative orientation in the Peruvian educational context since many teachers advising, evaluating, and/or coordinating research processes usually show specific threats, such as. *If I were a member of the thesis jury, I would disapprove of you*” and “*If you do not take my instructions into account, then I will stop* supporting *you*,” attitudes and behaviors that intimidate and generate fear and uncertainty in students. However, at this point, it is also necessary to clarify that some behaviors and attitudes of advisors and evaluating juries may be oriented towards academic demands and excellence, which, in contexts such as the Peruvian one, may be misinterpreted because a large percentage of university students are not used to the level of demand required to develop work with scientific rigor [20].

Secondly, the authoritarian parental educational style. In this case, this style becomes one of the three proposed by the parental educational model (authoritarian, negligent, and democratic) [21]. Thus, an authoritarian teacher usually gives orders, imposes, and displays his academic authority, applying iron discipline in the teaching-learning process, generating a distant and uncommunicative relationship with the student [22]. Hernandez and Prieto [23] emphasize that the role of authority is fundamental to understanding abuse towards students. Therefore, abuse of authority can be made up of degrading behaviors (a) that humiliate and denigrate self-esteem through verbal contempt; dominant behaviors (b) that define control parameters limit or repress critical thinking; discriminatory behaviors, (c) that harm the student based on their sex, race, economic level; destabilizing behaviors, (d) that intimidate, produce fear and anxiety; distancing behaviors, (e) that imply rejection, lack of emotional support and indifference towards the student; and diverse behaviors, (f) which are teacher attitudes that negatively influence the teacher-student interaction experience.

It is in this scenario that the importance of having valid and reliable measurement instruments arises, with which the magnitude of the problem of mistreatment of students who carry out their research work can be accurately assessed. Faced with this, Peruvian researchers developed a measure (EMAT) made up of 20 items distributed in 3 factors (advisors, juries, and administrative), the same that has motivated various studies at the national level [17]. However, the scope of this instrument is still limited, given that its initial validation was carried out with health sciences students, so it is not yet known if its psychometric performance is also optimal with students from other areas such as business sciences, social, humanities, engineering, among others. Furthermore, as the authors point out in the published report, the psychometric properties of the EMAT must be considered initial evidence, so it is urgent to carry out another type of analysis to make the test more robust, such as factorial invariance and the analysis under an IRT (Item Response Theory) perspective.

Taking into account the limited scope of the EMAT, the methodological gap regarding measures to evaluate abuse in research contexts, and the need to evaluate the magnitude of the problem of abuse in university research processes, this research is justified, which aims to provide new evidence of the psychometric properties of the EMAT in Peruvian university students, from different professional careers, from procedures based on Classical Test Theory (CTT) and Item Response Theory (IRT). Specifically, evidence of validity was evaluated based on the internal structure, reliability, discrimination characteristics and difficulty, and measurement invariance according to sex.

## 2 Method

### 2.1 Design

It corresponds to an instrumental design and cross-sectional study [24] since the aim is to study the psychometric properties of a documentary measurement instrument, with data collected only once in the timeline.

The study population was the students who concluded their research process, leading to obtaining a professional degree at their respective universities. These could be in their last year or academic cycle of studies, or by default; they could be university graduates. In this case, in Peru, according to the new University Law 30,220, the average duration of studies in this country is 5, and in some cases, 6 years, equivalent to 10 or 12 academic cycles (especially in medicine). In total, the sample comprised 753 Peruvian university students of both sexes, selected through intentional non-probabilistic sampling, applying the following inclusion criteria: 1) having completed the thesis process and 2) having approved the thesis defense. Likewise, as exclusion criteria, university students who were still in the process of preparing their degree work or in pending procedures before the thesis defense were considered.

The participants came from health, humanities, engineering, business, and law faculties of private and state universities from the 3 regions of Peru: Coast, Mountains, and Selva. A total of 57.4% of the participants were women, and 42.6% were men. Most of the participants resided in the coastal region (55.5%), were enrolled at some type of engineering college (28.7%) and attended private universities (98.9%). Further details regarding the characteristics of the participants can be seen in Table 1.

**Table 1:** Characteristics of the study sample.

	n	%
Age (*M* ± *SD*)	21.86	5.58
Sex		
Male	321	42.6%
Female	432	57.4%
Region of origin		
Mountains	312	41.4%
Coast	418	55.5%
Jungle	23	3.1%
Department		
Health	261	34.7%
Humanities	80	10.6%
Engineering	216	28.7%
Business	119	15.8%
Law	77	10.2%
Type of university		
Private	745	98.9%
State	8	1.1%

### 2.2 Instruments

Thesis Advisor Abuse Scale (EMAT) [17]. The EMAT consists of 20 items divided into 3 factors (advisor, committee and administrative). The responses to items are provided in a Likert-type scale ranging from 1 to 4 (never or rarely, sometimes, often, and almost all the time).

### 2.3 Procedures

This study was approved by the ethics committee of the Universidad Peruana Unión (N° 2022-CEUPeU-015). Information was collected online through Google Forms between May and July 2022. The link was shared via social networks (Facebook and WhatsApp) and by email. The first part of the form discussed informed consent and the objective of the study, clearly specifying that participation was voluntary and anonymous and that the data were only for research purposes.

### 2.4 Data analysis

The diagonally weighted least squares with mean and variance corrected (WLSMV) estimator was used to perform the confirmatory factor analysis (CFA) because the items presented 4 response categories [25]. To evaluate the fit of the models, the RMSEA, SRMR, CFI and TLI indices were used. For the RMSEA and SRMR indices, values less than 0.08 were considered acceptable [26]. For the CFI and TLI, values greater than 0.95 were considered adequate [27]. Cronbach's alpha coefficient was used to evaluate the reliability of the scale (Cronbach, 1951), as was the omega coefficient [28]; ꞷ>0.80 was considered adequate [29].

Regarding the IRT, an extension of the 2-parameter logistic model (2-PLM) was used for ordered polytomous items [30]. To estimate the fit of the models, the C2 test was used [31], and the following fit criteria were used: RMSEA ≤0.08 and SRMSR ≤0.05 [32]. The CFI and TLI values were also taken into account using the same fit criteria (≥0.95) used in CFA models, as suggested by the scientific literature [33]. Regarding the parameters of the items, the discrimination index (a) and difficulty level (b) were used. In addition, 3 thresholds were estimated for parameter b because the items have 4 response categories. Characteristic curves for the items (ICC) and the scale (TCC) were also calculated.

To evaluate the factorial invariance of the scale on the basis of sex, multigroup confirmatory factor analysis (MGCFA) was used, where a sequence of 4 hierarchical models of variance was proposed: (1) configural invariance (reference model), (2) metric invariance (equality of factorial loads), (3) scalar invariance (equality of factorial load and intercept) and (4) strict invariance (equality of factorial loads, intercept and residuals). To compare the sequence of models, a modeling strategy was used, for which the differences in the RMSEA (ΔRMSEA) were used, where differences less than <0.015 indicated invariance of the model between the groups [34].

For the statistical analysis, the RStudio environment was used [35] for R [36]. Specifically, the “lavaan” package was used [37] to perform CFA, the “semTools” package was used to perform the factorial invariance [37] and the “mirt” package was used for the IRT models [37].

## 3 Results

### 3.1 Descriptive analysis

The mean, standard deviation, skewness, and kurtosis of all items EMAT are seen in Table 2. It can be seen that item 2 (“I have felt obliged to obey all your instructions”) presents the highest average score in the sample. It is also seen that item 10 (“I have felt humiliated by your comments or attitudes regarding my thesis”) presents the lowest average score. It is observed that all items had skewness and kurtosis values of <2 and < 7, respectively, certifying the univariate normality of the data.

**Table 2:** Descriptive analysis of the scale items.

Items	*M*	*SD*	*g*1	*g*2
1. I felt that I couldn't contradict him/her/them because he/she/they did not listen to my opinions	1.69	0.84	1.04	0.25
2. I felt like I had to obey all his/her/their instructions	1.85	0.89	0.72	−0.45
3. He/she/they made offensive comments about my thesis	1.42	0.77	1.83	2.44
4. I felt that my ideas were belittled	1.46	0.79	1.66	1.86
5. I felt that the effort I made in the progress of my thesis was not noticed	1.51	0.81	1.52	1.45
6. When I tried to communicate, it took a long time for him/her/them to answer me	1.67	0.89	1.19	0.45
7. I felt little support in my work from him/her/them	1.62	0.87	1.24	0.52
8. I felt that I could not complain for fear of retaliation on the day I defend my thesis.	1.58	0.81	1.33	1.06
9. I felt like I needed to heed of all his/her/their observations, despite disagreeing	1.64	0.86	1.20	0.53
10. I felt humiliated by his/her/their comments or attitudes regarding my thesis	1.41	0.77	1.87	2.64
11. Even just the thought of having to approach him/her/them scared me	1.50	0.82	1.61	1.76
12. I felt that he/she/they did not appreciate all the effort that I put into my research work	1.51	0.81	1.58	1.78
13. It was difficult to communicate with him/her/them, as he/she/they took a long time to respond to my messages	1.56	0.83	1.38	1.00
14. I felt that they hindered my thesis more than supported it.	1.47	0.80	1.63	1.72
15. I felt that those in charge of processing my thesis documents did not care about how I felt	1.51	0.79	1.54	1.66
16. I felt that the research coordinator/manager did not care about my situation	1.50	0.81	1.59	1.68
17. The thesis process took longer than it should	1.51	0.81	1.51	1.38
18. When I needed procedural support with my thesis, I felt that the university did not provide it	1.51	0.81	1.54	1.52
19. I felt little empathy from those in charge of the research process to be able to advance with the procedures	1.50	0.82	1.59	1.63
20. I felt that the deadlines estimated in the research guidelines were not respected	1.48	0.81	1.67	1.90

Fig. 2 shows the characteristic curves for the items and dimensions (ICC and TCC).

### 3.2 Validity based on internal structure

**Fig. 1:**
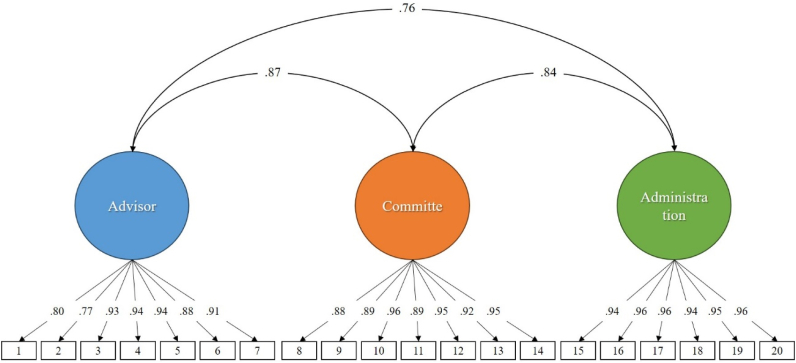
Confirmatory factor analysis of the scale.

A confirmatory factor analysis (CFA) was performed to test the previously proposed original factor structure of the EMAT. The original model of three related dimensions showed adequate fit indices (χ^2^ = 551.99; df = 167; CFI = 0.99; TLI = 0.99; RMSEA = 0.055 [90%CI 0.050 ‒ 0.061]; SRMR = 0.022). Fig. 1 shows the results of the CFA where all the standardized factor loadings of the items are high (>0.75), ranging between 0.77 and 0.96. All factor loadings were also statistically significant (p < 0.001). This shows that the items adequately represent the dimension to which they belong. The results suggest that the three-factor structure of the EMAT fits the data well. Therefore, this model was used in the following psychometric analyses.

### 3.3 Scale reliability

In the total study sample, the Advisor (α = 0.92; ω = 0.94), Committee (α = 0.94; ω = 0.95) and Administrative (α = 0.96; ω = 0.96) dimensions presented adequate index reliability. Similar results were obtained for men [Advisor (α = 0.93; ω = 0.94) Committee (α = 0.95; ω = 0.96) and Administrative (α = 0.97; ω = 0.97)] and for women [Advisor (α = 0.91; ω = 0.94) Committee (α = 0.94; ω = 0.95) and Administrative (α = 0.95; ω = 0.96)]. All these results indicate excellent reliability due to the internal consistency of the EMAT.

### 3.4 Item calibration with the gradual response model (GRM) (2-PML)

Two GRMs were fitted, specifically a 2PLM model for each dimension of the scale. As seen in Table 3, all the discrimination parameters for the items of the Advisor, Committee and Administrative dimensions were greater than 1, ranging between 1.82 and 3.84 generally considered good discrimination. Therefore, all items are very informative. Regarding the difficulty parameters, in the three dimensions, all the threshold estimators increased monotonically, as expected. The item parameters suggest that the third threshold (b4) is higher for items 1, 2, 3, and 4, which correspond to the advisory factor. This means that choosing the response option “almost all the time” for items 1, 2, 3, and 4 would indicate a higher level of mistreatment of the counselee than choosing the same response category for the rest of the EMAT items.

**Table 3:** Parameters of the items of the GRM models for the dimensions of the scale.

Dimension	Item	a	b_1_	b_2_	b_3_
Advisor	1	2.16	−0.01	1.29	2.60
	2	1.82	−0.35	1.06	2.59
	3	3.31	0.71	1.46	2.57
	4	3.49	0.53	1.38	2.57
	5	3.59	0.47	1.32	2.39
	6	2.56	0.20	1.33	2.05
	7	2.93	0.26	1.11	2.23
Committee	8	2.93	0.15	0.86	2.30
	9	2.71	0.10	1.02	2.23
	10	3.60	0.58	1.14	2.10
	11	2.96	0.41	0.98	1.81
	12	3.84	0.32	0.86	1.61
	13	3.10	0.27	0.90	2.18
	14	3.72	0.44	0.97	2.04
Administrative	15	3.61	0.08	1.28	2.03
	16	3.71	0.18	1.35	1.99
	17	3.25	0.13	1.16	2.04
	18	3.41	0.14	1.32	2.34
	19	3.71	0.23	1.13	2.05
	20	3.67	0.29	1.11	2.17

*Note.* A = discrimination parameter; b = difficulty parameter.

**Fig. 2:**
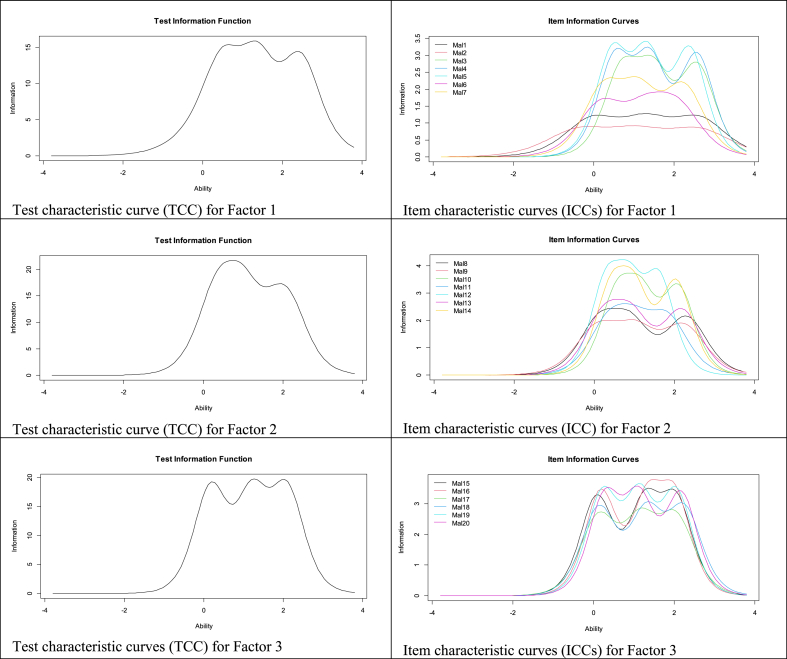
Item and test characteristic curves for the scale.

Fig. 2 shows the Characteristic Curves for the items and dimensions (ICC and CCT, respectively). Regarding the Advisor dimension, the ICC indicated that Items 4 and 5 were the most accurate for evaluating the latent trait. In addition, the TCC indicated that the factors were more reliable (accurate) in the range of the scale between 0 and 3. Regarding the Committee dimension, the ICC indicated that Items 12 and 14 were the most accurate for evaluating the latent trait. In addition, the TCC indicated that the factors were more reliable (accurate) in the range of the scale between 0 and 2.5. Finally, in the Administrative dimension, the ICC indicated that Item 16 was the most accurate to evaluate the latent trait. In addition, the TCC indicated that the factors were more reliable (accurate) in the range of the scale between −0.5 and 2.5.

### 3.5 Factorial invariance

Finally, to test the measurement invariance of the EMAT across gender, a series of multigroup CFAs were performed. Table 4 shows that configural invariance was fully compatible (RMSEA = 0.035; CFI = 0.93; SRMR = 0.030 according to gender. This allowed us to estimate models with increasing levels of restrictions to test higher levels of invariance. The factorial structure of the scale has shown evidence of being strictly invariant for the groups of men and women in the sequence of invariance models proposed: metric invariance (ΔRMSEA = −0.004), scalar (ΔRMSEA = −0.001) and strict (ΔRMSEA = −0.001). This finding suggested that both sexes had the same basic conceptualization of the EMAT and interpreted the items of each factor similarly.

**Table 4:** Model fit indices on the basis of sex and invariance indices.

One-dimensional model	χ^2^	df	*p*	SRMR	TLI	CFI	RMSEA [90% CI]	Δχ^2^	Δdf	*p*	ΔRMSEA
Women	346.72	167	0.000	0.029	0.99	0.99	0.050 [0.043 ‒ 0.057]	–	–	–	–
Men	434.29	167	0.000	0.028	0.99	0.99	0.071 [0.063 ‒ 0.079]	–	–	–	–
Invariance models											
Configural	490.72	334	0.000	0.030	0.93	0.93	0.035 [0.028 ‒ 0.042]	–	–	–	–
Metric	477.28	351	0.000	0.037	0.94	0.94	0.031 [0.024 ‒ 0.038]	25.84	17	0.077	−0.004
Scalar	491.71	368	0.000	0.038	0.95	0.94	0.030 [0.023 ‒ 0.037]	16.83	17	0.466	−0.001
Strict	512.98	388	0.000	0.041	0.94	0.94	0.029 [0.022 ‒ 0.036]	29.54	20	0.078	−0.001

Note: χ2 = chi square; df = degrees of freedom; SRMR: standardized root mean square residual; TLI = Tucker-Lewis index; CFI = comparative fit index; RMSEA = root mean square error of approximation; Δχ2 = change in chi square; Δdf = change in degrees of freedom; ΔRMSEA = change in root mean square error of approximation; ΔCFI = change in comparative fix index.

## 4 Discussion

The objective of this study was to provide new evidence, from the perspective of TCT and IRT, of the psychometric properties of the EMAT in Peruvian university students enrolled in different professional degrees. To date, the psychometric properties of the EMAT have been analyzed using TCT procedures. In this sense, this study provides one of the first empirical tests of the psychometric properties of EMAT from the perspective of IRT [38,39].

CFA revealed 3 related factors within the EMAT, lending empirical support to the factorial structure found in the original study [17]. Likewise, significant and relatively high factor loadings (>0.75) were reported. This provides more evidence that all the items of the EMAT are adequate indicators of the construct of the mistreatment of advisees and that the scale has a solid factorial structure. At a theoretical level, the finding confirms that the mistreatment of advisees occurs in relation to all the actors in the thesis process, such as the advisor, the committee and the administration. In this sense, advisors guide scientific production [40], and the function of the committee is to supervise the quality of the research [41,42]. However, in Peru, the scientific output of thesis advisors and members of examining committees is low [[43], [44], [45]]. This could lead to the exercise of power and hostility [1]. The administration guides students through the documentation process, which can be cumbersome and affect the mood of students [46]. The results of this study showed that the EMAT is highly reliable in its 3 dimensions, for both the subsamples of men and women. Thus, the EMAT is highly accurate in measuring mistreatment toward thesis students.

According to the IRT analysis results, all the items of the EMAT had good discriminatory power. Within the Advisory factor, Item 4 (“I felt that my ideas were belittled”) and Item 5 (“I felt that the effort I made in the progress of my thesis was not noticed”) were the most discriminative (that is, these items better distinguished between people with low and high levels of mistreatment) and the most accurate in the evaluation of this factor. These items indicate the presence of contempt and little appreciation for the work by thesis students, thus not favor horizontality or active participation by the student and the advisor. In Peru, advisors often use methods based on abuse and domination to teach their students [47]. This would have a negative impact on thesis students as advisors try to guide these individuals in the acquisition of skills to build knowledge and assume responsibility for their work. For the Committee factor, Items 12 (“I felt that he/she/they did not appreciated all the effort that I put into my research work”) and 14 (“I felt that he/she/they hindered my thesis more than supported it”) were the most discriminating and accurate. Similar to that for the Advisory factor, the low appraisal of effort by the examining committee would be considered an important indicator of mistreatment. The same occurs with the perception of a lack of support. This result is expected because it has been reported that committees can often be perceived as incoherent and mocking [48]. Similarly, they may exhibit negative attitudes, such as responding defiantly or ignoring communication requests, i.e., forms of psychological and academic mistreatment of students [49]. For the Administrative factor, Item 16 (“I felt that the research coordinator/manager did not care about my situation”) had the best discriminatory power and was the most accurate. This would suggest that for students who conduct a thesis, ignoring or rejecting their work on the part of the administrative staff would be an important indicator of mistreatment [1]. Finally, the monotonic increase in the difficulty parameters indicated that a greater presence of the latent trait (that is, mistreatment) is required to respond using the higher response categories of the EMAT.

The original psychometric analysis of the EMAT [17] did not detect the presence of measurement invariance; therefore, this was the first study to evaluate the ability to meaningfully compare groups using this scale. Furthermore, the study provided evidence that the three-factor structure of the EMAT was equivalent (or invariable) in 2 different subsamples, i.e., men and women. These findings indicate that the EMAT has a consistent factorial structure and can be interpreted in a similar way in different subsamples of men and women. As such, guidance counselors (psychologists), researchers and educational policy-makers can use the EMAT to better understand the differences between subsamples with regard to the perceived mistreatment of thesis students by advisors, examining committees and administrative staff. In other words, using the EMAT, it is possible to compare subsamples regarding the mistreatment by thesis advisors and, subsequently, design more effective prevention and intervention programs to address mistreatment by thesis advisors and its consequences. Importantly, men have reported more mistreatment during the development of their thesis than have women [1].

This study has limitations that should be considered when interpreting the results. First, the findings cannot be generalized due to the use of a nonprobability convenience sampling method. In this sense, it is necessary to have a more representative and diverse sample of the students to be able to compare and generalize the findings to other regions of Latin America and the world. A second limitation is the use of a self-report method to obtain data. This method generates social desirability biases as well as other method biases. Third, we only examined the factorial structure, discriminatory power and difficulty level of the items and the invariance of the EMAT. Therefore, no other additional psychometric analyses were performed, for example, test-retest reliability (which limits the knowledge regarding the stability of the scores), responsiveness or evidence of divergent and convergent validity. Fourth, some characteristics of the university students were not comparable in terms of distribution by gender, region of residence, or type of university. Therefore, for example, the test of measurement invariance between men and women could be somewhat biased. However, different demographic characteristics, such as gender, tend to lead to differences in outcome measures, making it likely that the invariance findings are underestimated rather than overestimated. Therefore, there is a higher probability that the invariance results are valid.

## 5 Conclusions

The results indicate that the EMAT has solid psychometric properties, on the basis of results generated using classical and modern statistical techniques, and serves as an adequate measure of mistreatment toward thesis students in Peruvian universities. Therefore, the EMAT can be used to help develop public policies aimed at directly addressing the mistreatment of advisees. In this sense, the EMAT allows the collection of information that supports actions to prevent long-term adverse consequences from mistreatment during thesis advising as well as guide interventions to target mistreatment during the thesis process. Likewise, the information collected with the EMAT can be used to help better understand and explore the sources of mistreatment toward thesis students. Finally, the EMAT places the symptoms of mistreatment as a particular source of distress within the specific context of the thesis advising process.

## Funding statement

This research did not receive any specific grant from funding agencies in the public, commercial, or not-for-profit sectors.

## Additional information

No additional information is available for this paper.

## Data availability statement

The datasets used and/or analyzed during the current study are available from the corresponding author on reasonable request.

## CRediT authorship contribution statement

**Oscar Mamani-Benito:** Writing – original draft, Validation, Supervision, Project administration, Investigation, Formal analysis, Conceptualization. **Maria Elena Rojas-Zegarra:** Writing – review & editing, Visualization, Validation, Supervision, Investigation. **Renzo Felipe Carranza Esteban:** Writing – original draft, Validation, Software, Investigation, Formal analysis. **Tomás Caycho-Rodríguez:** Writing – original draft, Visualization, Validation, Methodology, Investigation, Formal analysis, Data curation. **Lindsey W. Vilca:** Writing – original draft, Visualization, Validation, Methodology, Formal analysis, Data curation. **Susana K. Lingan:** Writing – review & editing, Visualization, Validation, Resources, Investigation.

## Declaration of competing interest

The authors declare the following financial interests/personal relationships which may be considered as potential competing interestsOscar Mamani-Benito reports article publishing charges was provided by University of the Lord of Sipan. If there are other authors, they declare that they have no known competing financial interests or personal relationships that could have appeared to influence the work reported in this paper.
