# Supplementary material for: New psychometric evidence for the thesis advisor abuse scale (EMAT) in Peruvian university students based on classic and modern procedures
Source: Heliyon. 2024 Mar 20;10(7):e28475. doi: 10.1016/j.heliyon.2024.e28475 (PMC10979084; doi:10.1016/j.heliyon.2024.e28475)
Supplement: Multimedia component 1 [file mmc1.docx]

**Thesis Advisor Abuse Scale (EMAT) / Escala de maltrato hacia el asesorado de tesis (EMAT)**

| items | Never or rarely / *Nunca o raras ocasiones* | Sometimes /*Algunas veces* | Often / *A menudo* | Almost all the time / *Casi todo el tiempo* |
| --- | --- | --- | --- | --- |
| 1. I felt that I couldn’t contradict him/her/them because he/she/they did not listen to my opinions/*He sentido que no podía contradecirlo, porque no escuchaba mis opiniones.* |  |  |  |  |
| 2. I felt like I had to obey all his/her/their instructions*/Me he sentido obligado a obedecer todas sus indicaciones* |  |  |  |  |
| 3. He/she/they made offensive comments about my thesis/*Ha hecho comentarios ofensivos acerca de mi tesis* |  |  |  |  |
| 4. I felt that my ideas were belittled/ *He sentido que ha menospreciado mis ideas* |  |  |  |  |
| 5. I felt that the effort I made in the progress of my thesis was not noticed/ *He sentido que no ha valorado el esfuerzo que he hecho en los avances de mi tesis* |  |  |  |  |
| 6. When I tried to communicate, it took a long time for him/her/them to answer me/ *Cuando he intentado comunicarme con él/ella, tardaba mucho en responderme* |  |  |  |  |
| 7. I felt little support in my work from him/her/them / *He sentido poco apoyo en mi trabajo de su parte* |  |  |  |  |
| 8. I felt that I could not complain for fear of retaliation on the day I defend my thesis/ *He sentido que no podía hacer un reclamo por temor a represalias el día de la sustentación* |  |  |  |  |
| 9. I felt like I needed to heed of all his/her/their observations, despite disagreeing /*Me he sentido obligado a hacer caso a todas sus observaciones, a pesar de no estar de acuerdo* |  |  |  |  |
| 10. I felt humiliated by his/her/their comments or attitudes regarding my thesis / *Me he sentido humillado(a) por sus comentarios o actitudes respecto a mi tesis* |  |  |  |  |
| 11. Even just the thought of having to approach him/her/them scared me / *Con tan solo pensar que aun debo acercarme a ellos, siento miedo* |  |  |  |  |
| 12. I felt that he/she/they did not appreciate all the effort that I put into my research work / *He sentido que no han valorado todo el esfuerzo que le he puesto a mi trabajo de investigación* |  |  |  |  |
| 13. It was difficult to communicate with him/her/them, as he/she/they took a long time to respond to my messages / *Ha sido difícil tener que comunicarme con ellos, pues tardaban mucho en responder mis mensajes* |  |  |  |  |
| 14. I felt that they hindered my thesis more than supported it / *Más que apoyo, he sentido que han puesto trabas a mi tesis* |  |  |  |  |
| 15. I felt that those in charge of processing my thesis documents did not care about how I felt / *He sentido que a los encargados de tramitar los documentos de mi tesis no les importa cómo me siento* |  |  |  |  |
| 16. I felt that the research coordinator/manager did not care about my situation / *He sentido que al coordinador/gestor de investigación no le ha importado mi situación* |  |  |  |  |
| 17. The thesis process took longer than it should /*El proceso de tesis se ha demorado más de lo debido* |  |  |  |  |
| 18. When I needed procedural support with my thesis, I felt that the university did not provide it / *Cuando he necesitado apoyo con los trámites de mi tesis, he sentido que la universidad no me ha dado las facilidades* |  |  |  |  |
| 19. I felt little empathy from those in charge of the research process to be able to advance with the procedures / *He sentido poca empatía por parte de los encargados del proceso de investigación para poder avanzar con los trámites* |  |  |  |  |
| 20. I felt that the deadlines estimated in the research guidelines were not respected / *He sentido que no se ha respetado los plazos estimados en el reglamento de investigación* |  |  |  |  |
